# Remimazolam besylate for the sedation of postoperative patients undergoing invasive mechanical ventilation in the ICU: a prospective dose‒response study

**DOI:** 10.1038/s41598-022-20946-6

**Published:** 2022-11-08

**Authors:** Xiaoyan Chen, Jiancheng Zhang, Shiying Yuan, Haiyan Huang

**Affiliations:** 1 Department of Critical Care Medicine, Institute of Anesthesia and Critical Care Medicine, Union Hospital, Tongji Medical College, Huazhong University of Science and Technology, 1277 Jiefang Avenue, Wuhan, 430022 China; 2 Institute of Anesthesia and Critical Care Medicine, Union Hospital, Tongji Medical College, Huazhong University of Science and Technology, Wuhan, 430022 China

**Keywords:** Clinical pharmacology, Drug safety

## Abstract

This single-center study aimed to determine the effective dose and safety of remimazolam besylate for the sedation of postoperative patients undergoing invasive mechanical ventilation in the intensive care unit (ICU). Mechanically ventilated patients admitted to the ICU after surgery were included. The Narcotrend index (NTI) was used to assess the depth of sedation, and the Richmond Agitation-Sedation Scale (RASS) score was also recorded. Remimazolam besylate was administered initially at a loading dose of 0.02 mg/kg, followed by a gradual increase of 0.005 mg/kg each time until the targeted depth of sedation was achieved (NTI 65–94). A maintenance dose of remimazolam besylate was administered starting at 0.2 mg/kg/h, followed by increments or subtractions of 0.05 mg/kg/h each time until a satisfactory depth of sedation was achieved and maintained for at least 30 min. The demographic data, anesthesia, surgery types, hemodynamics and respiratory parameters were recorded. Adverse events and adverse drug reactions were monitored for safety. Twenty-three patients were eventually included in this study covering a period of 1 year. A satisfactory depth of sedation was achieved by a single intravenous infusion of remimazolam besylate at a loading dose of 0.02–0.05 mg/kg followed by a maintenance dose of 0.20–0.35 mg/kg/h. There were no significant changes in hemodynamic and respiratory parameters within 10 min after the administration of remimazolam besylate. In addition, a significant correlation was observed between the NTI and the RASS score for assessing sedation (r = 0.721, *P* < 0.001). The NTI showed a predictive probability for a RASS score of 0.817. Remimazolam besylate was effective for mild/moderate sedation of invasively mechanically ventilated postoperative patients in the ICU while maintaining excellent respiratory and hemodynamic stability. The NTI can be used as a good tool for the objective evaluation of the depth of sedation and agitation.

## Introduction

Critically ill patients suffer from both physiological and psychological disorders, including but not limited to pain, agitation and anxiety, which are related to adverse clinical outcomes^1^. In addition to analgesics, clinicians generally use sedatives to help patients reduce anxiety, discomfort from mechanical ventilation, and complications related to agitation.

The pharmacokinetics of an ideal sedative for critically ill patients should be short-acting and organ-independent. There are many sedatives currently used in the intensive care unit (ICU), including propofol, midazolam, and dexmedetomidine, but none of these medications are ideal. Although propofol has the advantages of rapid onset and offset of action, it causes dose-dependent respiratory depression and hypotension due to systemic vasodilation. Additionally, it may cause injection pain and propofol infusion syndrome (PRIS)^2,3^. Midazolam has a good sedative effect and yields little cardiopulmonary instability. Moreover, the sedative effects of midazolam can be reversed by flumazenil. However, midazolam produces active metabolites, which may accumulate with prolonged administration^4^. Dexmedetomidine can induce sedation similar to physiological sleep^5^. Patients sedated with dexmedetomidine are more easily arousable and interactive, with minimal respiratory depression^6^. However, dexmedetomidine acts slowly, and it is difficult to achieve a satisfactory depth of sedation; moreover, the drug has side effects such as hypotension and bradycardia^7^.

Remimazolam is an ultrashort-acting intravenous benzodiazepine sedative that has been approved for use in anesthesia and procedural sedation in recent years with excellent respiratory and hemodynamic stability^8^. Remimazolam combines the properties of midazolam and remifentanil. It can be rapidly metabolized by tissue esterases independent of organs while retaining the pharmacological effects of benzodiazepines, which can produce good sedative effects^9^. Remimazolam is a promising sedative for ICU patients, but there is limited evidence of its effective dose and safety in the ICU. Moreover, studies have reported that in animal experiments, remimazolam can improve the survival rate of mice with LPS-induced endotoxemia^10^ and reduce sepsis-associated acute liver injury^11^. Therefore, it is speculated that the application of remimazolam for the sedation of patients with systemic inflammatory responses in the ICU may have potential benefits.

This study was performed in postoperative patients admitted to the ICU immediately after surgery with invasive mechanical ventilation to determine the effective dose and safety of remimazolam besylate for sedation and agitation.

## Patients and methods

### Study design and participants

**Figure 1:**
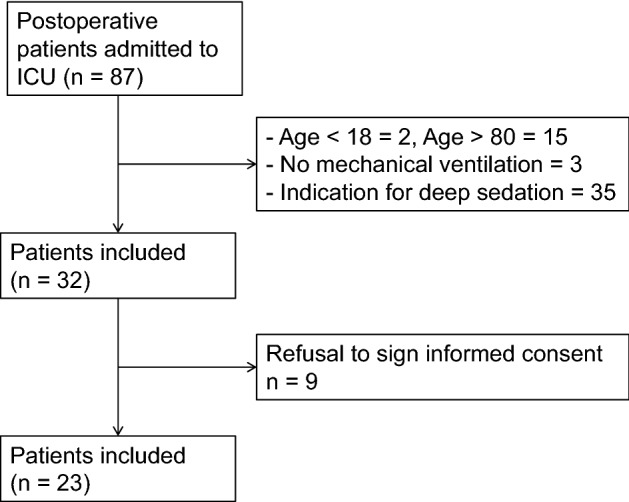
Enrollment flow diagram. Twenty-three postoperative patients with invasive mechanical ventilation in the intensive care unit (ICU) were finally included in the study.

This prospective dose‒response study was conducted in a multidisciplinary ICU with 26 beds. Eighty-seven postoperative patients admitted to the integrated ICU of Union Hospital, Tongji Medical College, Huazhong University of Science and Technology between March 31, 2020, and September 30, 2021, were screened using the following inclusion and exclusion criteria. The inclusion criteria included (1) patients aged between 18 and 80 years old and (2) patients admitted to the ICU immediately after surgery with general anesthesia and invasive mechanical ventilation. The exclusion criteria included (1) allergy to benzodiazepines; (2) myasthenia gravis and coma (Glasgow Coma Scale (GCS) score < 8); (3) pregnant or lactating women; (4) patients with uncorrected hemorrhagic or cardiogenic shock; (5) body mass index (BMI) < 18 kg/m^2^, or > 35 kg/m^2^; (6) chronic renal failure or chronic liver damage; (7) alcohol dependence, drug use, mental illness or severe cognitive impairment; and (8) requirement for deep sedation due to clinical considerations (RASS < –3). A total of 23 patients met these criteria and were included in the study (Fig. 1).

### Sedation assessment and interventions

In this study, the Narcotrend index (NTI) was used to assess the depth of sedation, and the Richmond Agitation-Sedation Scale (RASS) was also recorded to better titrate the dose of remimazolam within 24 h of admission to the ICU after surgery.

The NTI is an automatic electroencephalogram (EEG) recording system designed to assess the depth of sedation and anesthesia^12^, which has been proven to be equivalent to the bispectral index (BIS)^13–15^. The skin on the patient’s forehead was prepared with alcohol gauze to keep impedances less than 4 kΩ, and then three electrode pads were placed on the forehead and then connected to the machine with a distance of at least 8 cm between any two electrodes. The Narcotrend (MonitorTechnick, Bad Bramstedt, Germany) was used to assess the level of sedation: NTI 0–12 (burst suppression), 13–36 (deep anesthesia), 37–64 (general anesthesia), 65–79 (moderate sedation), 80–94 (light sedation), 95–100 (awake).

The RASS is a 10-point subjective scale designed to assess the level of sedation and agitation; the protocol is easy to perform and requires minimal training. The RASS has been widely used in ICU patients, and its reliability and validity have already been confirmed^16^. Five negative scores are assigned by level of tranquility (–5 = unarousable, –4 = deep sedation, –3 = moderate sedation, –2 = light sedation, –1 = drowsy), a score of 0 is assigned to patients who are alert and calm, and 4 positive scores are assigned by level of agitation (+ 1 = restless, + 2 = agitated, + 3 = very agitated, + 4 = combative).

After inclusion, the researchers confirmed that the patient was connected to an ECG monitor and placed protective restraints. At that time, any other sedatives the patients had taken were stopped, but the analgesics were retained (if necessary, sufentanil or nalbuphine were used to make the patient achieve an effective level of analgesia with the target of a score of zero on the Critical-care Pain Observation Tool (CPOT). After confirming that the patient could breathe spontaneously, the researcher adjusted the ventilator to a constant positive airway pressure (CPAP) + pressure support ventilation (PSV) mode.

Before the administration of remimazolam besylate, the patients should be awake from general anesthesia (NTI > 95, RASS score of 0 – + 1). The remimazolam besylate (36 mg, Jiangsu Hengrui Pharmaceutical Co., Ltd.) was formulated into 1 mg/ml with 0.9% saline and administered with a microinjection pump. The infusion doses of remimazolam besylate were calculated using the theoretical ideal weight of the patient [X + 0.91 (height–152.4), where X is 50.0 for men and 45.5 for women]. The loading dose of remimazolam besylate started from 0.02 mg/kg, which was injected within one minute. A supplementary bolus of 0.005 mg/kg remimazolam was administered until a RASS score of –3––1 and NTI value of 65–94 were obtained with the lowest possible dose, and the cumulative dose of remimazolam was recorded. The initial maintenance dose of remimazolam besylate was 0.2 mg/kg/h, followed by increments or subtractions of 0.05 mg/kg/h each time until a RASS score of –3––1 and NTI value of 65–94 were achieved, which was maintained for 30–120 min. The maximum dose was 0.1 mg/kg for the loading dose and 1 mg/kg/h for the maintenance dose. If the patients were still not effectively sedated when the maximum dose was reached, they would withdraw from the study and receive a rescue sedation regimen. During our study, if the RASS and NTI were significantly inconsistent, the RASS was the main reference. Adverse events and adverse drug reactions were monitored for safety.

### Data collection

The clinical data of the patients involved in this study included the following: (1) basic demographic data: age, sex, height, weight, body mass index, education level, comorbidities, etc.; (2) anesthesia and surgical data: American Society of Anesthesiologists (ASA) class, type of surgery, total surgical procedure time, total anesthesia time, anesthetics used during anesthesia, relaxants used during anesthesia, postoperative temperature, net fluid volume during surgery, and blood gas analysis before the study period; (3) monitored parameters: systolic blood pressure (SBP), diastolic blood pressure (DBP), mean arterial pressure (MAP), heart rate (HR), respiratory rate (RR), tidal volume (Vt), and oxygen saturation (SpO_2_); and (4) usage and drip speed of remimazolam. All data were checked by two researchers.

### Outcomes

The primary outcomes were the optimal dose of remimazolam besylate for a single intravenous infusion and subsequent continuous pumping. Secondary outcomes were the changes in hemodynamics and respiratory parameters (SBP, DBP, MAP, HR, RR, Vt, and SpO_2_) before and after the administration of remimazolam.

### Statistical analysis

Categorical variables were described as numbers (%). Continuous variables were described using the mean (SD) if they were normally distributed or the median (interquartile range, IQR) if they were not. The data were compared using one-way repeated-measures ANOVA. The correlation of the Narcotrend Index and the RASS was analyzed by Spearman’s related coefficient, and the prediction probability of the NTI for the RASS score was calculated by PKMACRO software based on the statistical approach proposed by Smith et al.^17^. *P* < 0.05 was considered statistically significant. The data collected were all analyzed using SPSS version 20.0 software (SPSS, Tokyo, Japan).

### Ethical approval and consent to participate

This research complies with the guidelines for human studies and was conducted ethically in accordance with the World Medical Association Declaration of Helsinki. Legal representatives provided written informed consent, and the study protocol was approved by the Ethics Committee of Union Hospital, Tongji Medical College, Huazhong University of Science and Technology (Permission number: 0445-01). This study was registered with the Chinese Clinical Trial Registry: ChiCTR2100043752, date of registration: 27/02/2021.

## Results

In total, 87 patients admitted to Union Hospital, Tongji Medical College, Huazhong University of Science and Technology between March 31, 2020, and September 30, 2021, were included in the study. Subsequently, we excluded the following patients: 35 patients who needed deep sedation, 15 patients aged more than 80 years, 2 patients aged less than 18 years, 3 patients who did not receive mechanical ventilation, and 9 patients whose legal representatives refused to participate in this trial (Fig. 1). Therefore, we included 23 patients in the final analysis. Their characteristics and medical information are shown in Table 1. These patients were between 51 and 72 years old, of whom 16 were males and 7 were females. Most of these patients had chronic diseases, including hypertension (43.5%), chronic heart disease (39.1%) and endocrine system disease (34.8%). They were enrolled in this study after an average of 261 (190–336) minutes of anesthesia and 222 (170–305) minutes of the surgical procedure. All the patients included were given propofol (3.85–5.85 mg/kg/h) and remifentanil (0.30–0.55 mg/kg/h) during anesthesia maintenance, which were discontinued before inclusion. Cisatracurium (73.9%) or rocuronium bromide (26.1%) was used as a muscle relaxant during anesthesia intubation, and the drug was discontinued before patients were admitted to the ICU. The median net fluid volume during surgery of these patients was 1800 (1200–2600) ml. The blood gas analysis before the study period of each patient is also recorded in Table 1.

**Table 1:** Patient characteristics.

	Total (n = 23)
Sex (male/female)	16/7
Age, median (IQR), years	63 (51–72)
< 50 years	3 (13.0)
50–65 years	9 (39.1)
≥ 65 years	11 (47.8)
Height, median (IQR), cm	170 (163–175)
Weight, median (IQR), kg	66 (61–73)
Ideal weight, median (IQR), kg	62 (55–67)
**Surgical site**	
Head and neck	6 (26.1)
Chest	2 (8.7)
Upper abdomen	2 (8.7)
Lower abdomen	7 (30.4)
Limbs	3 (13.0)
Urinary system	2 (8.7)
Endocrine organs	1 (4.3)
Total surgical procedure time, median (IQR), min	222 (170–305)
Total anesthesia time, median (IQR), min	261 (190–336)
**Anesthetics used during anesthesia, median (IQR), mg/kg/h**	
Propofol	4.92 (3.85–5.85)
Remifentanil	0.46 (0.30–0.55)
**Relaxants used during anesthesia**	
Cisatracurium	17 (73.9)
Rocuronium Bromide	6 (26.1)
Postoperative temperature, median (IQR), ℃	36.2 (35.8–36.8)
Net fluid volume during surgery, median (IQR), ml	1800 (1200–2600)
**Blood gas analysis before study period, median (IQR)**	
pH	7.383 (7.325–7.402)
PaO_2_/FiO_2_, mmHg	307 (229–441)
PaCO_2_, mmHg	41 (34.6–48.4)
Blood glucose, mmol/L	6.7 (5.2–9.1)
Hemoglobin, g/dL	11.5 (10.6–14.3)
CPOT score during study period, median (IQR)	0 (0–1)
**Comorbidities**	
Hypertension	10 (43.5)
Chronic heart disease	9 (39.1)
Endocrine system disease	8 (34.8)
Smoking	7 (30.4)
Digestive disease	6 (26.1)
Chronic respiratory disease	3 (13.0)
Disease of immune system	2 (8.7)
Hematological system disease	1 (4.3)

Values are numbers (percentages) unless stated otherwise.

IQR, interquartile range; n, number; min, minutes.

**Figure 2:**
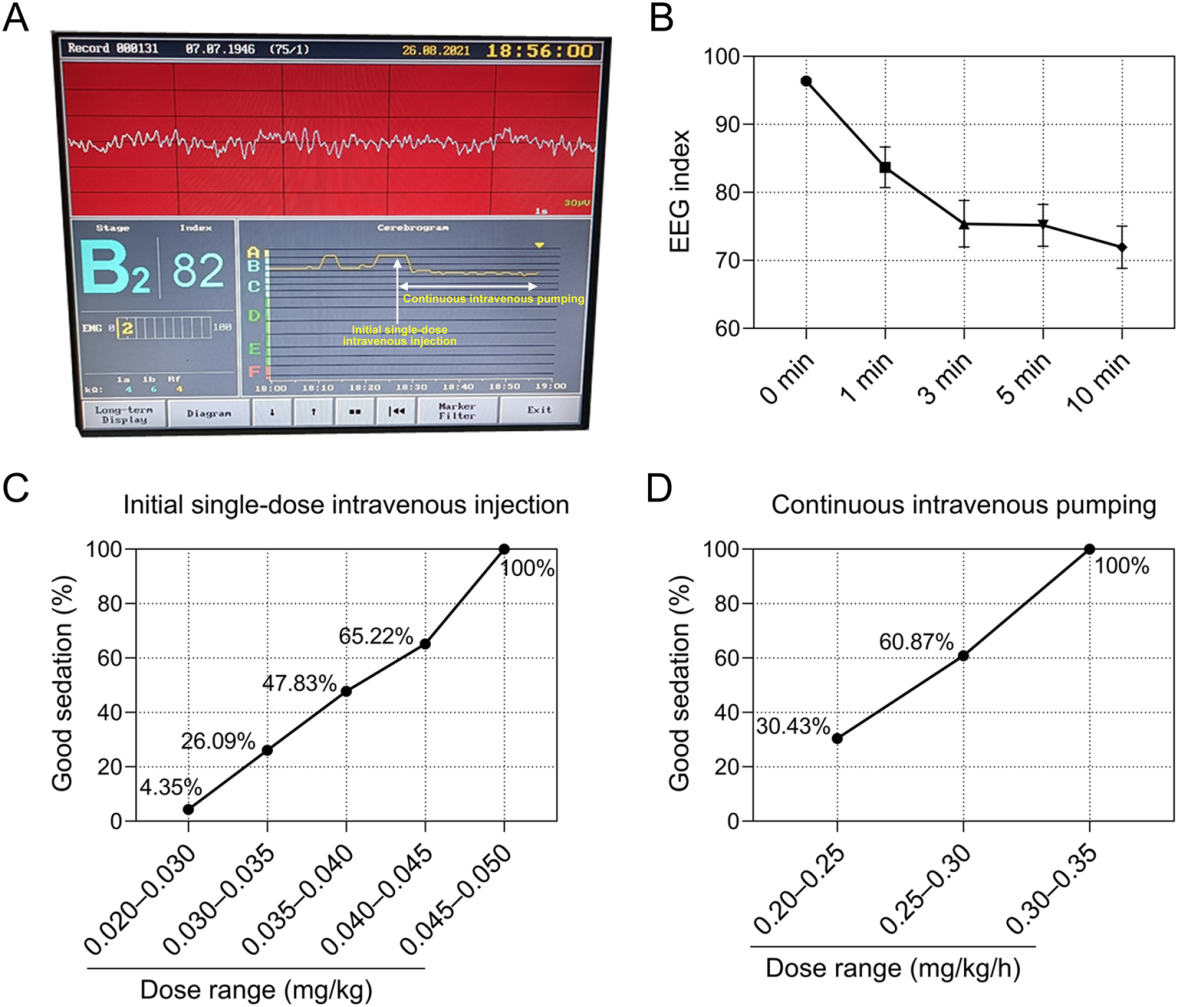
The optimal dosing of remimazolam besylate for a single intravenous infusion and subsequent optimal dosing for continuous pumping. (**A**) The Narcotrend monitoring view of one patient; the real-time electroencephalogram (EEG) and Narcotrend index (NTI) before and after the administration of remimazolam besylate. (**B**) The change in the average NTI of 23 patients at 0, 1, 3, 5, and 10 min after the administration of remimazolam besylate. Good sedation rates were achieved at different ranges of initial loading doses (**C**) and subsequent maintenance doses (**D**). Good sedation was defined as a RASS score of –3––1 and an NTI value of 65–94.

**Figure 3:**
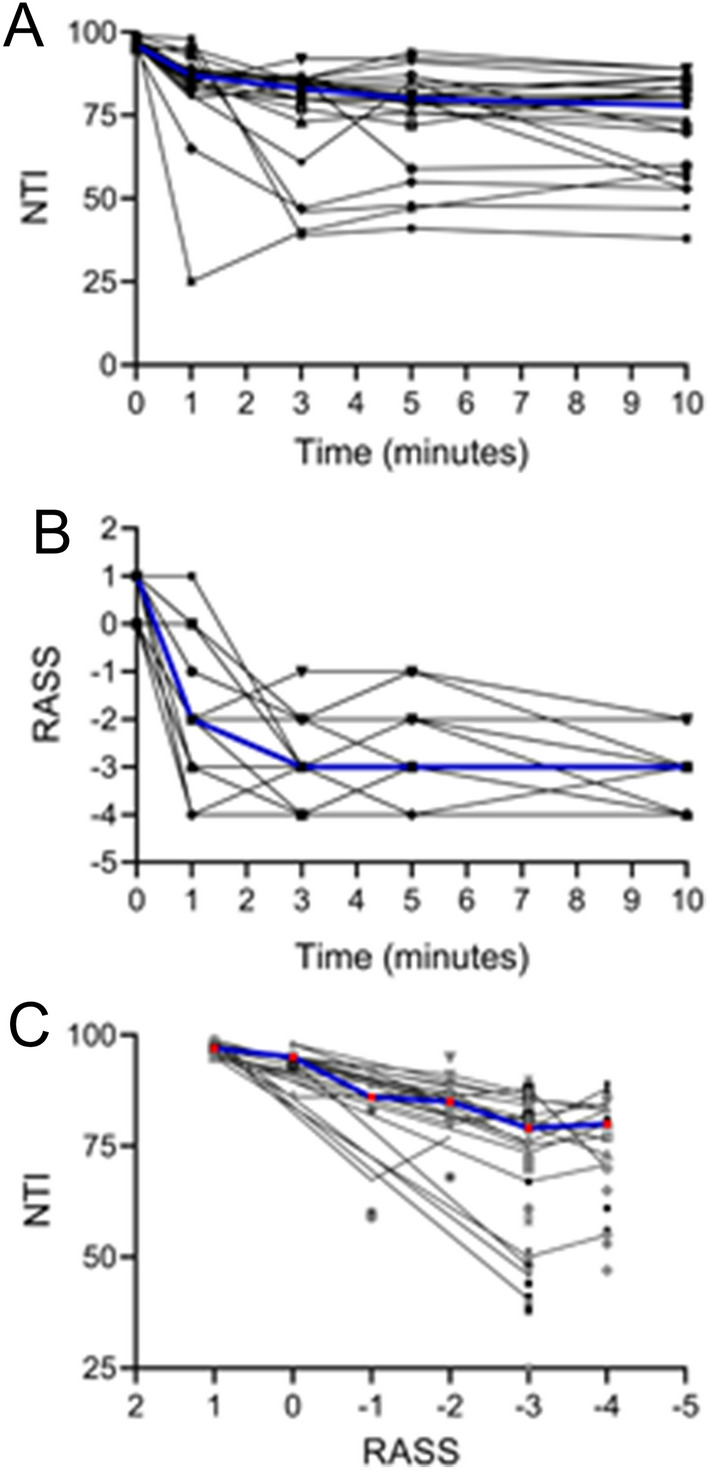
The change in the Narcotrend index (NTI; **A**) and Richmond Agitation-Sedation Scale (RASS; **B**) at 0, 1, 3, 5, and 10 min after the administration of remimazolam besylate. NTI and the corresponding RASS for each patient (**C**). The blue curve represents the median value.

Figure 2A shows the patient's Nyquist monitoring view. Figure 2B shows the change in the average NTI of 23 patients at 0, 1, 3, 5, and 10 min after administration. Although we set a range of values for the NTI to match the depth of sedation with reference to the instructions of the Narcotrend monitor, we found that in many cases, this range was not always applicable. The median baseline value of the NTI was 96 before the start of the remimazolam besylate infusion. The minimum NTI achieved during the first 10 min is characterized by large interindividual variation, with values ranging from 25 to 89 (Fig. 3). However, overall, the Spearman correlation coefficient for the NTI and RASS was 0.721 (*P* < 0.001), indicating a positive relationship between them. This was supported by the prediction probability, which ranged from 0.564 to 1 in each patient, and the overall prediction probability value for the RASS was 0.817.

The loading dose was initially administered from 0.02 mg/kg, and the bolus was completed within six minutes. The 0 min at the beginning of the abscissa in Fig. 3A,B refers to the time of the last supplemental dose before the patient reached the sedation target as assessed by the RASS score and NTI. All patients included achieved good sedation when given a loading dose of 0.02–0.05 mg/kg (Fig. 2C) and a maintenance dose of 0.20–0.35 mg/kg/h (Fig. 2D).

The changes in hemodynamics and respiratory parameters of the patients at 0, 1, 3, 5, and 10 min after the administration of remimazolam besylate are shown in Table 2. There were no significant differences in the changes in SBP, DBP, MAP, HR, RR, Vt, or SpO_2_. During our study, no adverse events or adverse drug reactions were recorded. One patient received vasoconstrictor drugs to maintain blood pressure due to a decrease (MAP dropped from 85 to 61 mmHg in 10 min) during the study, and the other patients did not take rescue measures due to changes in vital signs.

**Table 2:** Hemodynamics and respiratory parameters during sedation.

	0 min	1 min	3 min	5 min	10 min
SBP	141 ± 24	139 ± 22	136 ± 23	133 ± 26	129 ± 24
DBP	72 ± 11	71 ± 11	70 ± 11	68 ± 11	66 ± 10
MAP	95 ± 14	95 ± 14	92 ± 14	89 ± 14	86 ± 13
HR	85 ± 20	85 ± 20	85 ± 21	85 ± 22	83 ± 20
RR	16 ± 6	14 ± 6	14 ± 5	13 ± 4	13 ± 3
Vt	621 ± 153	565 ± 109	569 ± 114	566 ± 103	583 ± 90
SpO_2_	99 ± 2	99 ± 2	99 ± 2	99 ± 2	99 ± 2

Systolic blood pressure (SBP, in mmHg), diastolic blood pressure (DBP, in mmHg), mean arterial pressure (MAP, in mmHg), heart rate (HR, in beats/min), respiratory rate (RR, in times/min), tidal volume (Vt, in mL), and oxygen saturation (SpO_2_, in %) are expressed as mean ± SD.

## Discussion

This study aimed to determine the optimal dose of remimazolam besylate for sedation in patients admitted to the ICU after surgery with invasive mechanical ventilation. The RASS and NTI were used to evaluate the depth of sedation at the same time. The results of this study showed that with a loading dose of 0.02–0.05 mg/kg remimazolam besylate and a subsequent maintenance dose of 0.20–0.35 mg/kg/h, satisfactory sedative effects was obtained without significant instability of respiratory and hemodynamic parameters.

ICU patients are prone to agitation and anxiety, which are related to many underlying factors, including pain, delirium, hypoxia, hypoglycemia and psychological factors, which may lead to adverse clinical outcomes^18^. Rational use of sedative drugs can help patients reduce anxiety and stress and promote compliance with mechanical ventilation and invasive procedures.

Remimazolam is a new type of sedative modified from midazolam that has high affinity and selectivity for γ-aminobutyric acid (GABA) receptors^8^. Additionally, with properties of benzodiazepines, remimazolam sedation can be reversed by flumazenil. This drug is synthesized from midazolam as the parent compound with the addition of an ester moiety, making it easy to hydrolyze by nonspecific tissue esterase enzymes, which means that remimazolam can be metabolized without the need for any particular organ^19,20^. This will make it a valuable drug for patients with hepatic or renal dysfunction. Remimazolam has shown good application prospects in previous clinical trials, and currently, Phase II and Phase III clinical trials are being carried out in various countries. In a clinical trial involving 62 healthy Chinese volunteers, the safety, pharmacokinetics and pharmacodynamics of remimazolam besylate were evaluated after a single administration and continuous infusion. Remimazolam is considered safe, well tolerated, and has predictable pharmacokinetic (PK) properties and dose-dependent pharmacodynamic (PD) properties. Compared with midazolam, remimazolam may have a stronger sedative effect and may yield a faster recovery from sedation^21^. In multiple phase II or phase III clinical trials^22–24^, compared with midazolam, remimazolam provided sufficient sedation in a range of endoscopic procedures, such as colonoscopy, upper gastrointestinal endoscopy, and bronchoscopy. High procedure success rates and good tolerance were observed in the remimazolam groups. Some clinical trials have confirmed that the effectiveness and safety of remimazolam in the induction and maintenance of general anesthesia are comparable to those of propofol ^25–27^. Currently, many phase III clinical trials of remimazolam for sedation in ICU patients are underway, but clinical data have not been reported.

In this study, the NTI and RASS were used to simultaneously assess the patient's sedation status to better titrate the dose of remimazolam. We found that the NTI was suitable for monitoring the patient’s state of clinical sedation and correlated well with the RASS score. The Pain, Agitation, and Delirium (PAD) guidelines in 2013 suggested that the RASS is one of the most effective and reliable sedation assessment tools for measuring the quality and depth of sedation in adult ICU patients^18^. Studies have shown that Narcotrend monitoring can reduce the use of sedative drugs and reduce drug-related complications^13,28^. Narcotrend is an automatic EEG analyzer that automatically converts the EEG into visual indicators by monitoring the patient’s original EEG and divides the person’s state of consciousness into six categories^12^. In many clinical studies, Narcotrend has been proven to be equivalent to BIS and can provide an effective reference in monitoring the depth of anesthesia and sedation^13,14^. However, in a clinical study where the NTI was used to monitor the depth of sedation following continuous infusion of remimazolam in healthy male volunteers, the NTI and Modified Observer's Assessment of Alertness and Sedation score did not show a consistent relationship, and the NTI appeared less suitable than the beta ratio for monitoring the sedative effect if remimazolam was administered alone^29^. Unlike this study, our study subjects were ICU patients using different dosing regimens, and there was no withdrawal wake-up process. Thus, the drug reaction and monitoring results of the patients would be different. Here, our study used the NTI to assist the RASS in the assessment of remimazolam sedation. Although the NTI is variable among patients and does not always match the RASS well to monitor the depth of sedation, our data showed that the overall prediction probability of the NTI for the RASS score was 0.8. To the best of our knowledge, this is the first study to use the NTI in the monitoring of remimazolam-sedated ICU patients. More large prospective clinical trials are needed to verify the efficacy of Narcotrend for monitoring the sedative effect of remimazolam in ICU patients.

The patients included in this study were surgical patients, most of whom had chronic comorbidities. Directly after surgery, such patients may be unstable due to factors such as suboptimal fluid balance, low body temperature, remaining anesthetic effects, etc. We recorded the net fluid volume during surgery, postoperative temperature and blood gas analysis before the study period (Table 1), and we found that most patients were in a stable condition and that few patients had hypothermia, volume depletion, anemia or metabolic acidosis. These factors will indeed affect the recovery process of patients and the effect of medication, but to what extent they may have affected our research is not clear and needs to be verified by further studies. During our study, no adverse events or adverse drug reactions were recorded. Only one patient used vasoactive drugs to maintain blood pressure due to a drop in blood pressure. It should be noted that although benzodiazepines have fewer cardiorespiratory inhibitory effects, vigilance is still needed when they are used for critically ill patients in the ICU. Because such patients often have complex medical conditions, the side effects may have unpredictable adverse outcomes.

The purpose of this study was to determine the optimal dose of remimazolam for postoperative patients admitted to the ICU. For the first time, we used the RASS combined with the NTI to evaluate the sedation of patients to titrate the dose of remimazolam besylate. However, this study still has many shortcomings. We did not set up a positive control group to compare the sedative effect of remimazolam, nor did we monitor its blood concentration. In addition, this was a single-center study with a small population. Moreover, our enrolled subjects were postoperative patients with less severe disease and cannot represent all patients in the ICU. Regarding the sedation of remimazolam in ICU patients, a large number of clinical trials are still needed for exploration.

## Conclusion

Remimazolam is a new type of benzodiazepine that has shown good application prospects in general anesthesia and procedural sedation. Phase II and Phase III clinical trials of remimazolam for sedation in ICU patients are currently underway. Our study shows that when remimazolam besylate is used for sedation of postoperative ICU patients receiving mechanical ventilation, a loading dose of 0.02–0.05 mg/kg and a subsequent maintenance dose of 0.20–0.35 mg/kg/h can obtain satisfactory sedative effects. At the same time, we are the first to use the RASS and NTI to evaluate remimazolam-sedated patients, providing a reference for the use of sedation assessment tools for ICU patients.

## Data Availability

The datasets used and/or analyzed during the current study are available from the corresponding author upon reasonable request.

## Abbreviations

ICU: Intensive care unit
NTI: Narcotrend index
RASS: Richmond Agitation-Sedation Scale
EEG: Electroencephalogram
SBP: Systolic blood pressure
DBP: Diastolic blood pressure
MAP: Mean arterial pressure
HR: Heart rate
RR: Respiratory rate
Vt: Tidal volume
SpO_2_: Oxygen saturation
